# DIA Comparative Proteomic Analysis of Retro-oil Fluid and Vitreous Fluid From Retinal Detachment Patients

**DOI:** 10.3389/fmolb.2021.763002

**Published:** 2021-12-03

**Authors:** Yiyang Shu, Min Gao, Yifan Zhou, Haiyun Liu, Xiaodong Sun

**Affiliations:** ^1^ Department of Ophthalmology, Shanghai General Hospital, Shanghai Jiao Tong University School of Medicine, Shanghai, China; ^2^ National Clinical Research Center for Eye Diseases, Shanghai, China; ^3^ Shanghai Key Laboratory of Ocular Fundus Diseases, Shanghai, China; ^4^ Shanghai Engineering Center for Visual Science and Photomedicine, Shanghai, China; ^5^ Shanghai Engineering Center for Precise Diagnosis and Treatment of Eye Diseases, Shanghai, China; ^6^ Putuo People’s Hospital, Tongji University, Shanghai, China

**Keywords:** data-independent acquisition (DIA), quantitative proteomics, silicone oil, rhegmatogenous retinal detachment (RRD), vitreous

## Abstract

**Objectives:** There have been reports of unexplained visual loss following intra-ocular silicone oil (SiO) tamponade in retinal detachment patients, yet the underlying mechanism is unknown. The aim of this study was to investigate the mechanisms behind retinal toxicity following intra-ocular SiO tamponade in retinal detachment patients.

**Methods and Results:** Vitreous fluid samples were acquired from 27 patients (27 eyes). Twelve eyes for data-independent acquisition (DIA) were divided into four groups: pars plana vitrectomy (PPV) for rhegmatogenous retinal detachment (RD group), SiO removal after successful retinal reattachment (SO group), cataract surgery after successful retinal reattachment with sterilized air tamponade (FA group), and PPV for epiretinal membrane (ERM group). The remaining 15 eyes were used for enzyme-linked immunosorbent assay analysis. DIA was combined with two-dimensional liquid chromatography–tandem mass spectrometry to find expression changes in the proteome of vitreous. Mean number mass spectra, statistically differentially expressed proteins, gene ontology (GO), pathway representations, and protein interactions were analyzed. GO analysis showed that the protein categories of synapse organization, cell adhesion, and regulation of cell migration in the SO group were differentially expressed compared to the control or FA groups (*p* < 0.05). Through Kyoto Encyclopedia of Genes and Genomes (KEGG) analysis, lysosome and cell adhesion were found to be significantly enriched in the SO group compared to the FA and control groups (*p* < 0.05). Cadherin 2, transferrin, and lysosome function may partially contribute to silicone oil-related vision loss.

**Conclusion:** Vision loss-inducing novel molecular signatures and pathways that may be associated with SiO toxicity were identified. Transferrin may be a potential visual outcome biomarker for SiO tamponade.

## Introduction

Rhegmatogenous retinal detachment (RRD) is an ocular emergency with an incidence of 10–55 per 100,000 individuals per year (Van de Put et al., 2013). One of the most popular and effective surgical options is pars plana vitrectomy (PPV), which could achieve high reattachment rates in RRD patients. Silicone oil (SiO) was frequently used intraocular tamponade during retinal repair, of which, due to the prolonged tamponade period, SiO could dramatically increase the success rate of retinal reattachment patients suffering from severe pathological fundus situations, such as giant retinal tears, severe proliferative retinopathy (PVR), recurrent detachment, and choroidal detachment (Cibis et al., 1962). However, a series of reports have indicated the presence of unexplained severe visual damage related to intravitreal SiO use, which is also known as silicone oil-related visual loss (Newsom et al., 2004; Cazabon et al., 2005; Herbert et al., 2006). To date, the underlying mechanism is still unclear.

Quantitative proteomics provides an effective approach to understand the global proteomic change by identifying and quantitatively comparing proteins in various diseases (Santos et al., 2018). Data-independent acquisition (DIA) is a new mass spectrometry technology developed in recent years (Zhang et al., 2020). DIA combines sequential isolation of precursor window and total production spectroscopic acquisition to sequence all detectable peptides in samples which provide the most comprehensive information. In fundus diseases, due to close contact and protein exchange between the vitreous and the inner retina, the proteome change of the vitreous body or vitreous fluid could provide clues to physiological and pathological conditions of retina disorders (Shitama et al., 2008). To our knowledge, published information about vitreous proteomics in SiO tamponade is scarce.

In the present study, we initially compared the quantitative proteomic profiling among vitreous fluids of RRD, epiretinal membrane, and retinal repair (SiO or sterilized air tamponade) patients by employing the combination of liquid chromatography–tandem mass spectrometry (LC–MS/MS) with a DIA workflow. We investigated the differences in proteomic enrichment data in these groups and further reported important insights into the possible pathological mechanisms of silicone oil tamponade in RRD patients.

## Material and Methods

### Patient Enrollment and Vitreous Fluid Collection

A total of 27 patients who undertook fundus surgery at the Department of Ophthalmology in Shanghai General Hospital were enrolled. Vitreous fluid was obtained from 12 patients during four different surgeries (each group, *n* = 3) for DIA proteomic analysis: PPV for RRD (RD group), SiO removal after successful retinal reattachment (SO group), cataract surgery after successful retinal reattachment with sterilized air tamponade (FA group), and PPV for epiretinal membrane (ERM group). The ERM group was regarded as control. For enzyme-linked immunosorbent assay (ELISA) analysis, vitreous fluid was obtained from 15 other patients (15 eyes: five FA eyes during cataract surgery for the ELISA-FA group and 10 SO eyes during SiO removal for the ELISA-SO group) to confirm the differentially expressed protein transferrin. Patients with metabolic diseases including hypertension and diabetes, ocular trauma, or any associated concurrent eye condition, such as vitreous hemorrhage, macular degeneration, high myopia, glaucoma, uveitis, or grade C to D PVR were excluded. The PVR was graded according to the updated classification of the Retina Society Terminology Committee (1991) (Machemer et al., 1991). Data concerning prior surgeries, duration of retinal detachment, date of primary retinal repair surgery, and severity of PVR were recorded as clinical characteristics.

Before intravitreal operation, undiluted samples of vitreous humor, retro-oil fluid, or vitreous cavity fluid were obtained under visual control by aspirating liquefied vitreous from the vitreous cavity with a 1-ml syringe connected to a hose flute needle before the infusion. The volume of samples is approximately 200–300 μl. Undiluted samples were divided into 1.5-ml tubes (Eppendorf, Freemont, CA, United States), frozen at −270°C immediately, and kept at −80°C until analysis.

### In-Solution Digestion and Peptide Fractionation for LC–MS/MS

The samples were digested, and the peptides were desalted and fractioned. The samples were mixed in 8 M urea, 100 mM Tris-HCl, pH 8.5, and 5 mM DTT, then 1 M iodoacetamide (15 mM IAA in UA buffer) was added to block reduced cysteine residues, and the samples were incubated for 40 min in darkness. Finally, 0.5 µg Lys-C (1:100) was added and set for 4 h, then 0.5 µg trypsin was added and set for 16 h at 37°C, and the resulting peptides were collected as a filtrate. The peptides of each sample were desalted and concentrated by vacuum centrifugation and reconstituted in 10 µl of 0.1% (v/v) formic acid. Furthermore, 2-μg sample peptides were mixed with some standard iRT peptides for further data-dependent acquisition (DDA) and DIA quantification analysis.

### LC–MS/MS DIA

LC–MS/MS DIA analyses were performed in cooperation with Applied Protein Technology (APTBIO, Shanghai). Shotgun data-dependent mass spectrometry and assay library generation were completed using 2 ug peptides that are label-free, mixed with iRT peptides, and analyzed using a Knauer Smartline HPLC system, Easy nLC-1200 (Fortis Technologies, Neston, United Kingdom), according to instructions, operating in shotgun mode for 2 h. Peptide fractionation was operated at a flow rate of 250 nl/min with 95% buffer A, followed by a linear increase to 30% buffer B across 97 min, to 100% B across 13 min, and was sustained at 100% for 10 min. The top 20 most intense MS1 precursors with charge states between 2 and 5 were used for MS2 fragmentation, with a 15-s exclusion window. Fragment MS2 ions were collected for 120 ms across a 300–1,800-m/z range. Peptide sequences from raw profile mode wiff files were assigned using parallel searches against UniProtKB databases of human proteins with Maxquant software (1.5.3.17), appended with common contaminants and decoy sequences. Precursor ion mass errors were set to 30–75 ppm.

DIA was analyzed. Similarly, samples and iRT peptides were label-free and analyzed using a Knauer Smartline HPLC system, Easy nLC-1200 (Fortis Technologies, Neston, United Kingdom), according to instructions, operating in shotgun mode for 2 h. Peptide fractionation was operated at a flow rate of 250 nl/min with 95% buffer A, followed by a linear increase to 30% buffer B across 97 min, to 100% B across 13 min, and was sustained at 100% for 10 min. Each DIA cycle contained one full MS–SIM scan, and 30 DIA scans covered a mass range of 350–1,650 m/z with the following settings: SIM full-scan resolution was 120,000 at 200 m/z; AGC 3e6; maximum IT 50ms; profile mode; DIA scans were set at a resolution of 30,000; AGC target 3e6; Max IT auto; and normalized collision energy was 30 eV.

### LC–MS/MS Data Analysis

For DDA library data, database was searched by using MaxQuant 1.5.3.17 software. The database was downloaded at the website http://www.uniprot.org. The iRT peptide sequence was added into the database (>Biognosys|iRTKit|Sequence_fusionLGGNEQVTRYILAGVENSKGTFIID PGGVIRGTFIIDPAAVIRGAGSSEPVT GLDAKTPVISGGPYEYRVEATFGVDES NAKTPVITGAPYEYRDGLDAASYYAPVRA DVTPADFSEWSKLFLQFGAQGSPFLK). The parameters were set as follows: enzyme, trypsin; maximum missed cleavage site, 2; fixed modification, carbamidomethyl (C); and dynamic modification, oxidation (M) and acetyl (Protein N-term). All reported data were based on 99% confidence for protein identification as determined by false discovery rate {FDR = *N* (decoy) * 2 / [*N* (decoy)+ N (target)]} ≤ 1%. A spectral library was constructed by importing the original raw files and DDA searching results into Spectronaut Pulsar X_12.0.20491.4 (Biognosys).

DIA data was analyzed with Spectronaut Pulsar X_12.0.20491.4 searching the above-mentioned constructed spectral library. The main software parameters were set as follows: retention time prediction type, dynamic iRT; interference on MS2 level correction, enabled; cross-run normalization, enabled. All results were filtered based on a *Q* value cutoff of 0.01 (equivalent to FDR <1%).

### Bioinformatic Analysis

Differentially expressed proteins were clustered by hierarchical cluster method, and data was presented as heat maps. Gene Ontology (GO) terms were analyzed, and the main process of GO analysis included blast, GO mapping, GO annotation, and interproScan for annotation augmentation. The differences of proteins were calculated using Fisher’s exact test. GO enrichment of differential proteins was studied for biological process, cellular component, and molecular function using Blast2Go (Software Tool, https://www.blast2go.com). Pathway enrichment of protein clusters was performed by using Kyoto Encyclopedia of Genes and Genomes (KEGG, http://www.kegg.jp/) pathway database. The differences of enrichment pathways were calculated using Fisher’s exact test. To assess functional associations between differential proteins, the online tool STRING tool (Search Tool for the Retrieval of Interacting Genes/Proteins) was applied with high confidence (0.70) and organism (*Homo sapiens*).

## Results

### Patient Demographics and Clinical Characteristics

The demographics and clinical characteristics of patients enrolled in the study and the description of the corresponding vitreous samples are summarized in Tables 1. The study cohorts consisted of 27 patients—14 women and 13 men—with ages between 52 and 77 years. The sample cohort for DIA comprised 58.3% men, and there was no significant difference in age and sex between groups (*p* > 0.05). Fifteen patients were selected for the validation of transferrin by ELISA with similar age and sex. Detached retina extended two to three quadrants without the involvement of macula in all RD patients. The most common ocular comorbidity were lattice degeneration and grade A to B PVR, which were balanced between groups.

**TABLE 1:** Demographics and clinical characteristics of the study population.

Demographics and clinical characteristics	SO	FA	ERM	RD	*P*	FA-ELISA	SO-ELISA	*P*
	*N* = 3	*N* = 3	*N* = 3	*N* = 3		*N* = 5	*N* = 10	
Sex, *N* (%)								
Males	1 (33.3)	2 (66.7)	2 (66.7)	2 (66.7)		2 (40.0)	4 (40.0)	
Females	2 (66.7)	1 (33.3)	1 (33.3)	1 (33.3)	1.0^a^	3 (60.0)	6 (60.0)	1.0^a^
Age, Y								
Median (range)	71 (60–77)	66 (60–70)	64 (58–69)	61 (55–68)	0.305^#^	64 (52–70)	62 (52–72)	0.728″
PVR grade								
A	1	1		1		1	1	
B	2	2		2		2	2	

p-value was calculated by ^#^Kruskal-Wallis H test, ^a^Chi-square test or ^″^Independent-samples T test.

SO, SO removal after successful retinal reattachment; FA, cataract surgery after successful retinal reattachment with sterilized air tamponade; ERM, PPV for epiretinal membrane; RD, PPV for rhegmatogenous retinal detachment; FA-ELISA, FA group for ELISA; SO-ELISA, SO group for ELISA; *N*, number; Y, years.

### Spectral Library Generation and LC–MS/MS DIA Data Extraction

**FIGURE 1:**
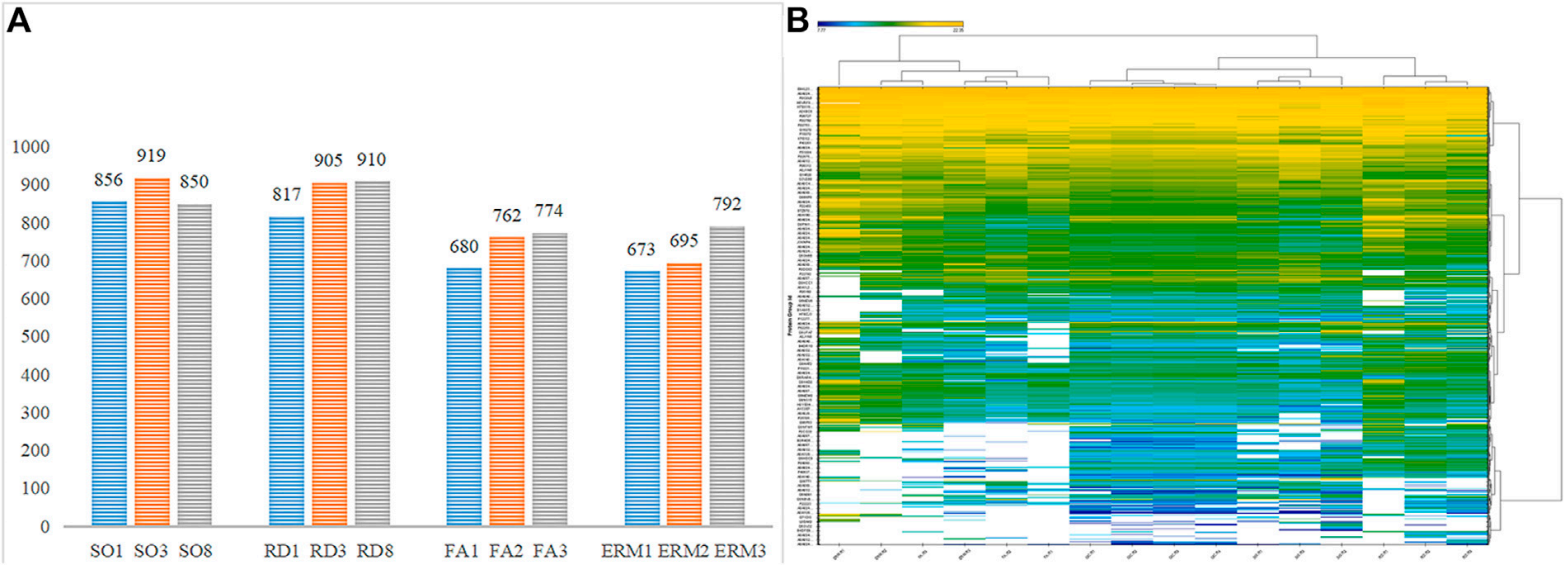
**(A)** The number of protein groups in the four groups. **(B)** Quantitative heatmap of data-independent acquisition.

The DDA analysis of fractionated peptides by HPLC system Easy nLC-1200 identified 1,016 protein groups and 6,768 peptides at a detection threshold of 0.05. The proteins (false discovery rate, FDR <0.01) and peptides were added to the spectral library for LC–MS/MS data identification and extraction. Each sample was then separately prepared, fractioned, and analyzed for DIA data extraction. With data imported into Sepctronaut Plusar X, different protein groups were identified and quantified following retention time alignment. Protein expression patterns were different between groups in protein distribution and expression content. It was found that there were 817, 905, and 910 protein groups for the three individuals in the RD group; 856, 919, and 850 protein groups for SO; 680, 762, and 774 for FA; and 673, 695, and 792 protein groups for ERM that acted as control. The number of protein groups of RD and SO samples was slightly larger than that of ERM or FA (Figure 1A). A heat map of clustered fold changes for the main differentially expressed proteins was presented (Figure 1B).

### Data Quality and Quantification Reproducibility

**FIGURE 2:**
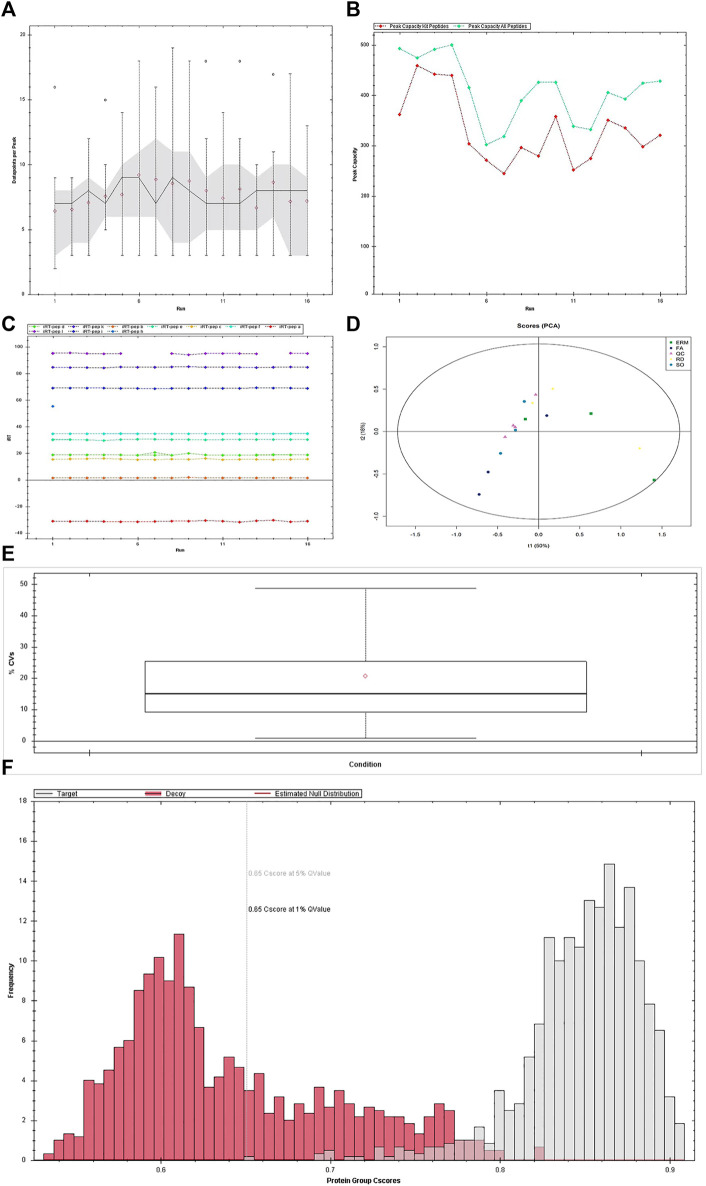
Quality control of data-independent acquisition. **(A)** Average data points per peak: the average data points per peak was 8.1, which met the requirements of the quantitative analysis. **(B)** Column peak capacity statistics: the x-axis presents the order of the samples, the green line details the data of all peptides, and the red one details the data of the iRT internal standard. Peak capacity represented the separation and analysis capability of the column. The average peak capacity was 409.8, indicating better separation and analysis. **(C)** Elution time of iRT peptide segment: the main iRTs were detected, and the retention time was generally stable. **(D)** Chart of principal component analysis (PCA). Detected changes in the protein shown in PCA plots could well explain the differences in this experiment. **(E)** Protein false discovery rate (FDR) distribution data. The qualitative results are highly reliable under the FDR 0.01 standard. **(F)** CV distribution of QC samples. The median CV of the QC sample was 15.1%, indicating the high stability of the experimental process.

The average data points per peak, peak capacity, and iRT stability were reproducible across the samples (Figures 2A–C). The principal component analysis plots, protein FDR, and coefficient of variance of the quality control samples using the top 50 differentially abundant proteins showed stability and reproducibility across all the samples (Figures 2D–F).

### Compare Vitreous Proteins Profiling in Four Groups

**FIGURE 3:**
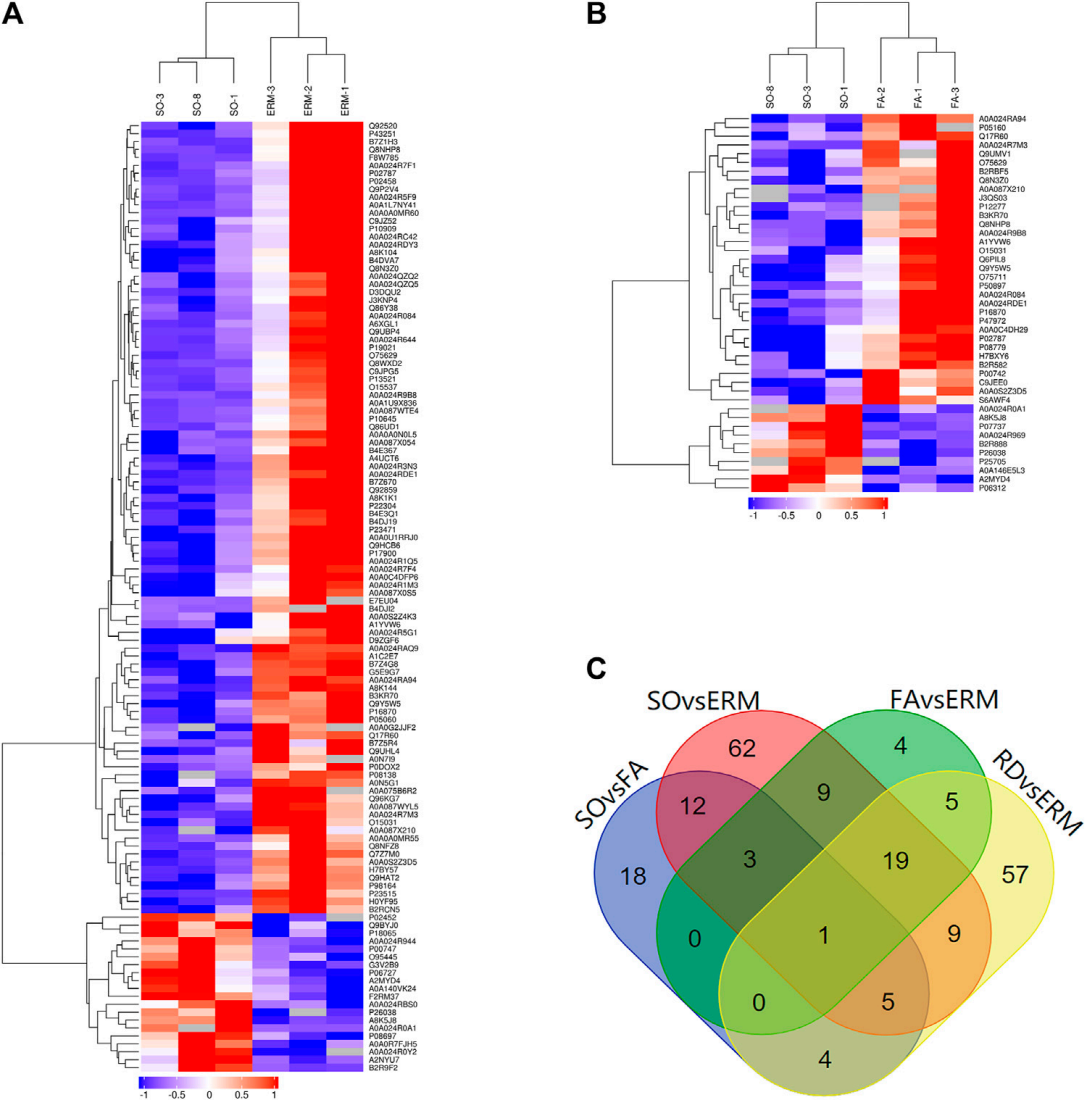
Differential protein clusters. **(A) **SO *vs*. ERM. **(B)** SO *vs*. FA. **(C)** The overlapping changes between four groups of RD, ERM, FA, and SO.

The differential abundance of proteins was analyzed using hierarchical cluster. From the identified proteins, 43 were found to be differentially expressed (10 upregulated and 33 downregulated proteins) in the SO group compared to the FA group (SO *vs*. FA), 120 (20 upregulated and 100 downregulated) in the SO group compared to the ERM group (SO *vs*. ERM), 100 (32 upregulated and 68 downregulated) in the RD group compared to the ERM group (RD *vs*. ERM); and 41 (five upregulated and 36 downregulated) in the FA group compared to the ERM group (FA *vs*. ERM) (fold change >1.5 or <−1.5 and *p*-value <0.05) (Table 2). Of these proteins, a heat map of clustered fold changes for the majority of differentially abundant proteins was summarized (Figures 3A,B). The overlapping changes between four groups of RD, ERM, FA, and SO were then shown in a Venn diagram (Figure 3C). Among the overlapping proteins, four protein groups were differentially abundant in all the groups, including profilin-1, coagulation factor X, immunoglobulin kappa variable 4-1, and immunoglobulin lambda-like polypeptide 1. Importantly, several differentially abundant proteins could be related to SiO treatment in analyzing SO *vs*. FA and SO *vs*. ERM profiling. The protein categories in the FA group were similar to the control.

**TABLE 2:** The number of differentially expressed proteins in each group.

Comparisons	Up-	Down-	All-
SO *vs*. FA	10	33	43
SO *vs*. ERM	20	100	120
RD *vs*. ERM	32	68	100
FA *vs*. ERM	5	36	41

Comparisons, differential comparison group; Up-, upregulated differentially expressed proteins; Down-, downregulated differentially expressed proteins; All-, all differentially expressed proteins; SO *vs*. FA, the SO group compared to the FA group; SO *vs*. ERM, the SO group compared to the ERM group; RD *vs*. ERM, the RD group compared to the ERM group; FA *vs*. ERM, the FA group compared to the ERM group.

### GO Enrichment Analysis

**FIGURE 4:**
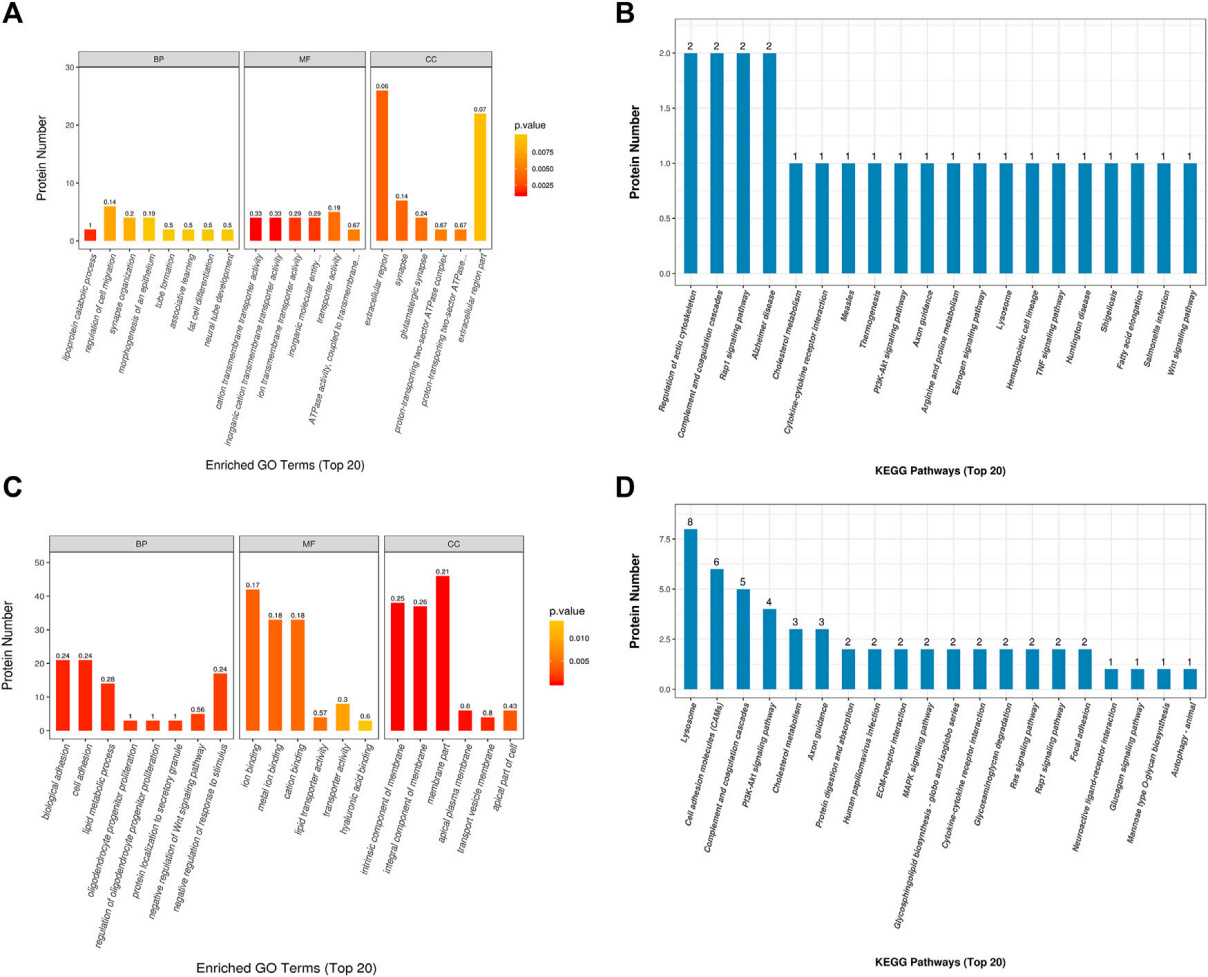
Gene Ontology and Kyoto Encyclopedia of Genes and Genomes analyses. **(A**, **B)** SO *vs*. FA. **(C**, **D)** SO *vs*. ERM.

The GO enrichment analysis of biological functions associated with the top 20 proteins which are differentially abundant in SO *vs*. FA showed that the SO group was enriched for proteins related to lipoprotein catabolic process, regulation of cell migration, synapse organization, morphogenesis of an epithelium, tube formation, associative learning, fat cell differentiation, and neural tube development (*p*-value <0.05). These proteins mainly functioned as cation transmembrane transporter, inorganic cation transmembrane transporter, iron transmembrane transporter, and in ATPase activity (*p*-value <0.05). They were mainly distributed in the extracellular region, synapse, glutamatergic synapse, and proton-transporting two-sector ATPase complex (*p*-value <0.05) (Figure 4A). The GO enrichment analysis of proteins differentially abundant in SO *vs*. REM showed that the SO group was enriched for proteins related to biological adhesion, cell adhesion, lipid metabolic process, negative regulation of response to stimulus, and negative regulation of Wnt signaling pathways (*p*-value <0.05). These proteins mainly play roles in iron binding, metal ion binding, cation binding, hyaluronic acid binding, transporter activity, and lipid transporter activity (*p*-value <0.05). The enriched proteins were mainly distributed in various components of the cell membrane, transport vesicle membrane, and apical part of cell (*p*-value <0.05) (Figure 4C).

The comparison analysis of RD *vs*. REM showed that the RD group was enriched for proteins related to cell recognition, immune response-regulating signaling pathway, immune response-activating signal transduction, antibacterial humoral response, and phagocytosis (*p*-value <0.05). These proteins mainly functioned in immunoglobulin receptor binding, glycosaminoglycan binding, signaling receptor binding, antigen binding, and hyaluronic acid binding (*p*-value <0.05). They were distributed in different cell components, such as membrane part, membrane, external side of the plasma membrane, cell surface, immunoglobulin complex, and circulation (*p*-value <0.05).

### KEGG Analysis and Protein–Protein Interaction Network Analyses

Using the KEGG pathway database, the differentially expressed proteins in the SO *vs*. FA and SO *vs*. ERM groups are shown in Figures 4B,D. Three pathways related to measles, fatty acid elongation, and hematopoietic cell lineage were significantly enriched in SO *vs*. FA. In addition, in the SO *vs*. ERM comparison, lysosomes and cell adhesion molecules were significantly enriched.

The KEGG analysis of the RD *vs*. ERM group revealed several main pathways associated with RD, including complement and coagulation cascades, cell adhesion molecules, platelet activation, glycolysis/gluconeogenesis, HIF-1 signaling, proteoglycans in cancer, antigen processing and presentation, microRNAs in cancer, and apoptosis. Among these, pathways related to cell adhesion molecules (CAMs), HIF-1 signaling pathway, platelet activation, propanoate metabolism, and glycolysis/gluconeogenesis were significantly enriched.

**FIGURE 5:**
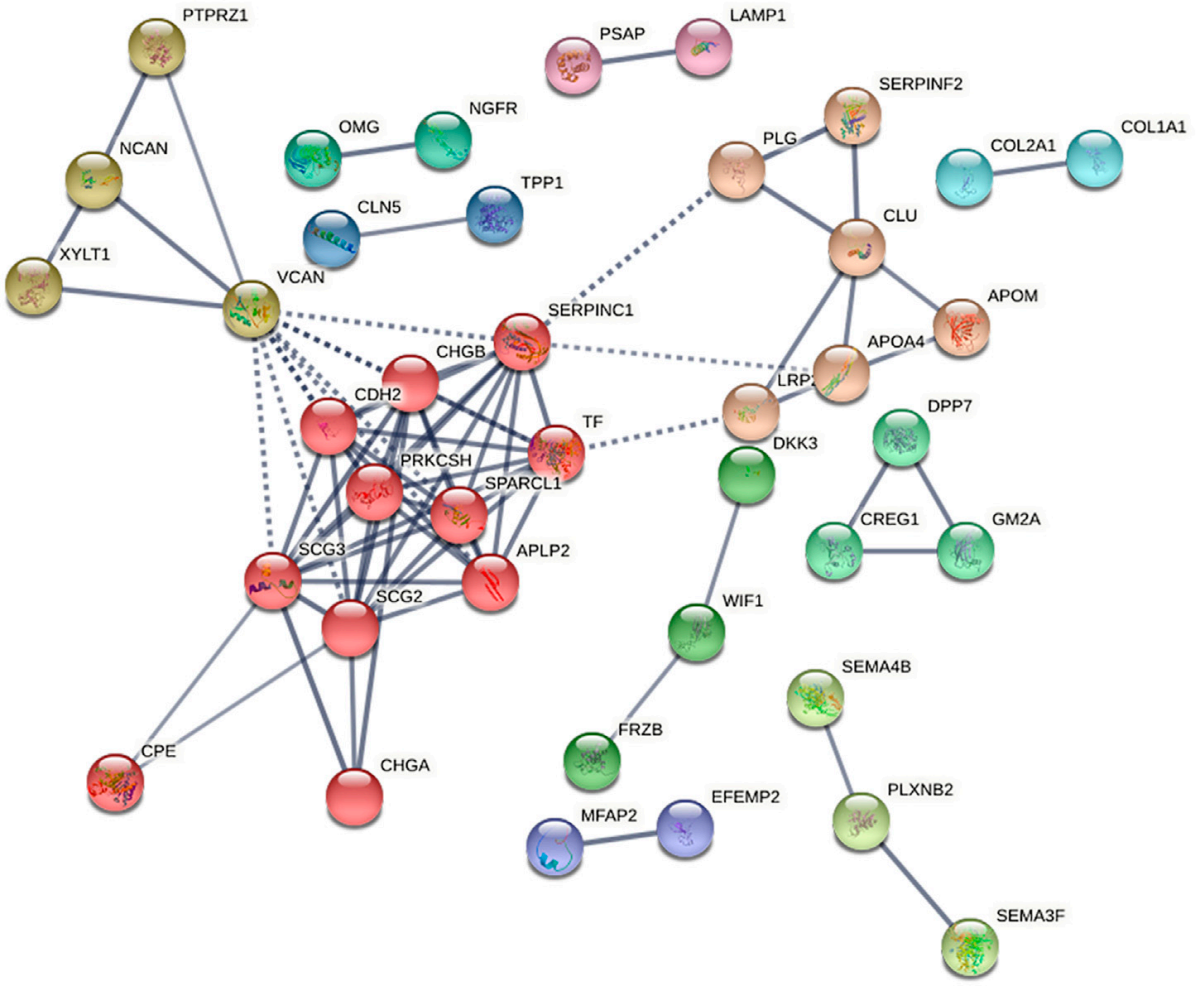
The protein–protein interaction network for differentially expressed proteins in SO *vs*. ERM, predicted by STRING 11.0 set to high confidence (0.70), organism (*Homo sapiens*), that is based on interaction evidence. The protein–protein interaction network was grouped into 11 relevant protein clusters using the Markov Cluster Algorithm (inflation parameter, 3) clustering option provided by STRING.

Protein–protein interaction network (PPI) analyses were then made by STRING database (https://string-db.org/) with high confidence (0.70) for four groups. The network is enriched in only two interactions between the total of 27 differential proteins in SO *vs*. FA with a PPI enrichment (*p*-value = 0.183). Furthermore, there were 78 interactions enriched between the 74 proteins found to be differentially expressed in SO *vs*. ERM with a PPI enrichment (*p*-value <1.0e-16). The PPI network was grouped into 11 relevant protein clusters using the Markov Cluster Algorithm (inflation parameter, 3) clustering option provided by STRING (Figure 5). These clusters share interactions among themselves, indicating that the molecules involved could play key roles in diverse pathways that contribute to SiO-related retinal cell pathology.

To infer the possible associated functions, clusters were classified according to Reactome pathways (Supplementary Tables S1, S2). According to the Reactome pathway analysis, cluster 1 (red) is larger and is associated with post-translational protein phosphorylation, regulation of insulin-like growth factor (IGF) transport, platelet degranulation, formation of fibrin clots, and metabolism of proteins. Cluster 1 (red) is composed of APLP2, CDH2, CHGA, CHGB, CPE, PRKCSH, SCG2, SCG3, SERPINC1, SPARCL1, and TF.

In addition, the PPI analysis of the RD *vs*. ERM group revealed 58 interactions to be enriched between the 54 proteins with a PPI enrichment (*p*-value <1.0e-16). Through the Reactome pathway analysis, one important and relevant protein cluster—cluster 1 (red)—is larger and associated with the regulation of post-translational protein phosphorylation, regulation of IGF transport, and glycosaminoglycan metabolism. In cluster 1 (red), the proteins included APLP2, GOLM1, HSP90B1, LUM, NCAN, PTPRZ1, SHISA5, SPARCL1, and VCAN.

### Validation by ELISA

**FIGURE 6:**
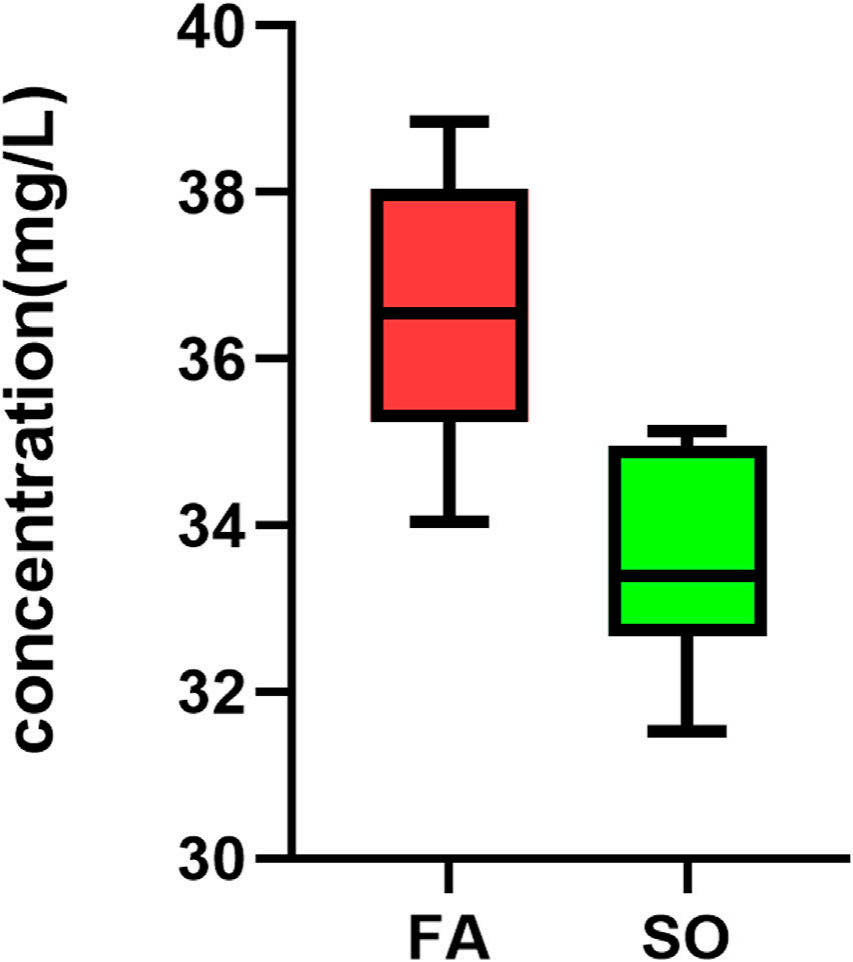
Box plots showing TF levels. To validate the finding, TF was measured with ELISA in Ten SO group and 5 FA group that were matched according to age and sex. TF was significantly downregulated in SO eyes relative to FA eyes (*p*-value = 0.002, independent-samples *t*-test; *n* = 15).

After the discovery-based proteomics, transferrin was found to be an important node involved in SO *vs*. FA and SO *vs*. ERM group. Owing to the biological significance and also to the availability of the experiment, the protein was chosen for further testing using ELISA (YX-E201806H, Sinobestbio). The amount of transferrin was determined in 15 samples, including 10 ELISA-SO and five ELISA-FA. For the 10 eyes in SO, a level of 33.56 ± 1.292 mg/L transferrin was found, with a range of 31.52–35.14 mg/L. For the five eyes in ELISA-FA, a level of 36.63 ± 1.73 mg/L transferrin was found, with a range of 34.05–38.85 mg/L. For ELISA confirmation, transferrin was significantly downregulated in SO eyes relative to FA eyes (*p* < 0.05) (Figure 6).

## Discussion

SiO is one kind of intravitreal tamponade in vitrectomy for retinal detachment. Concerns about visual impairment related to SiO tamponade have been raised by many surgeons. Proteomic analysis using DIA mass spectrometry is a powerful method to provide comprehensive information, which accounts for global protein expression with high accuracy. Using this platform, our data set demonstrated a significant protein expression differentiation in vitreous specimens among SO, FA, and control eyes. We also discovered the proteomic changes in the RD group compared to the control. The identification factors with differential expression suggested that protein categories of cell migration, synapse organization, and cell adhesion were related to SiO toxicity. We confirmed CAMs, HIF-1 signaling, and glycolysis metabolism changes after RD compared to the control.

Classical clinical specimen for proteomic analysis of ocular retinopathy includes the blood, aqueous humor, retinal tissue, and vitreous fluid of the patients (Pollreisz et al., 2013; Gopalakrishnan et al., 2015; Wang et al., 2019). Among these specimens, vitreous could directly suffer biochemical, proteomic, and fluid properties that change overtime, corresponding to the pathological state of the retina (Shitama et al., 2008). Thus, vitreous is a suitable approach for pathogenesis study. Based on this, recent studies have reported several proteomic profiles of vitreous fluid materials in various eye diseases, including retinal detachment, proliferative diabetic retinopathy, epiretinal gliosis, vitreoretinopathy, retinoblastoma, and inherited retinopathy (Aretz et al., 2013; Garcia-Ramirez et al., 2007; Gaspar et al., 2017; Naru et al., 2016; Yu et al., 2008). However, the number of publications about proteomic analysis in RRD was limited. Several studies using isobaric tags for relative and absolute quantitation (iTRAQ) labeling had reported more than 100 proteins differentially expressed in RRD vitreous fluid when compared with the control. They found activation of the HIF-1 pathway and accumulation of photoreceptor proteins in response to RRD (Santos et al., 2018). Furthermore, Wu *et al*. identified 103 proteins in RRD with choroidal detachment compared to RRD by iTRAQ–mass spectrometry (Wu et al., 2016).

DIA has become the technology of choice for the high-throughput characterization of proteins and proteomes. Unlike traditional DDA-based methods which acquire MS/MS (or MS2) data for selected precursor ions, DIA adopts different data scanning mode equations to acquire both MS1 and MS2 data without bias to the precursor ion selection and without omission or difference (Zhang et al., 2020). The DIA technique can collect all ion information to achieve higher data coverage and reduce the randomness of collection, resulting in a very high level of reproducibility and stability. The quantitative precision, accuracy, and linear range are greatly improved by using fragment ion quantification by DIA.

In the current study, we applied DIA with two-dimensional LC–MS/MS to compare the proteomic changes of vitreous fluid in the RD group to the control group of ERM. With KEGG enrichment analysis, HIF-1 signaling pathway and glycolysis metabolism were enriched in vitreous fluid from RD compared to the control, which was consistent with previous reports. In the glycolysis pathway, proteins ENO2, LDHA, and ALDOA were significantly upregulated, which was discussed before (Santos et al., 2018). We also identified cell adhesion molecule signaling differentiation, which has not been found in previous studies. Proteins neurexin, selectin, and neuronal cell adhesion molecule were significantly downregulated compared to the control. These proteins may be downregulated in RRD as a consequence of the disruption in photoreceptor–RPE connection, retina neuron cell adhesion, or RPE disorder after RRD.

Although SiO is used as intraocular tamponade, yet there is a risk of vision loss for RD surgery, either during tamponade or after SiO removal (Williams et al., 2008; Roca et al., 2017). Intraocular SiO leads to functional and morphological changes with different durations of tamponade after RRD vitrectomy, and the duration of SiO tamponade is positively correlated with the final visual acuity in RRD patients (Scheerlinck L. M. et al., 2016). Studies have reported retinal thinning and intra-retinal cysts to be linked to SiO tamponade and concluded that ganglion cell death and ellipsoid zone integrity are the reasons for vision loss (Shalchi et al., 2015; Scheerlinck L. M. et al., 2016). Some studies suggest that SiO-mediated retinal toxicity in retro-oil fluid could play a role. Retinal electrolytes were found to accumulate in SiO tamponade eyes by Scheerlinck *et al*. (2016). They concluded that LDH was an indicator of SiO-related damage. LDH was also found to be upregulated in retro-oil fluid, but there was no statistical difference compared to the FA or control groups. To better understand the mechanisms of SiO toxicity and visual loss, we collected the retro-oil fluid in SiO tamponade for proteomic analysis using DIA with LC–MS/MS.

First, we identified the enrichment of cell adhesion molecule signaling pathways in the SO group compared to the control, which had not yet been reported in previous studies. Proteins cadherin 2, neurexin, neuronal cell adhesion molecule, and versican core protein were significantly downregulated compared to the control. It suggested the existence of a disruption in retinal neuron cell connections that persisted after SiO tamponade through the retina which was reattached. Thus, SiO tamponade may affect and delay the recovery of retinal cell adhesion compared to the FA group.

Second, the lysosome pathway was enriched in the SO group. Proteins alpha-N-acetylgalactosaminidase, hexosaminidase, and iduronate 2-sulfatase, deoxyribonuclease II were slightly downregulated. It suggested that SiO tamponade could affect the lysosome metabolism and membrane integrity in retinal cells, which contributed to further retinal cell injury or photoreceptor autophagy (Santos et al., 2018). Studies reported that the defect of the autophagy–lysosomal pathway was related to RPE, photoreceptors, and inner retinal neuron degeneration in an experimental model (Hayasaka, 1983), yet the existence of lysosomal failure and its impact that resulted from SiO tamponade was still unexplored.

Transferrin was found to be significantly downregulated in the SO group compared to the control and FA group. According to previous reports, transferrin functions as iron clearance by binding to its glycoprotein transporter, which is involved in the regulation of ferroptosis and mineral absorption in the brain (Crichton et al., 2011; Eid et al., 2017). The excess intracellular iron could block the genomic repair system and promote neurodegeneration by ferroptosis. Iron induces immediate necrosis and delayed apoptosis. Our data shows that transferrin is downregulated in both the FA and SO groups, and the decrease is profound in the SO group. We speculate that both retinal detachment and SiO may have impacts on transferrin concentration. Previous reports have verified that transferrin can reduce both apoptosis and necroptosis and identified the pathway mediating the neuroprotective effect of transferrin after RD on mice (Daruich et al., 2019). Transferrin dysregulation may partially involve the intracellular iron concentration in SiO tamponade eye and RD eye. Thus, it is suggested that iron metabolism disorder in the retina could be one of the risk factors for the pathogenesis of SiO damage.

There are several limitations in this study. Although we analyzed 12 specimens, the sample size of each group was still relatively small. In addition, the proteomic analysis was based on the vitreous fluid but not the retinal cells or tissues. Although direct contact and exchange of active molecules exist between vitreous fluid and retina layers, proteins identified by proteomic analysis of vitreous fluid only partially reflect the biological change of retinal neurons in the eyes. Lastly, even though the study was conducted using DIA that can achieve high accuracy, further studies under laboratory conditions are still needed to verify that these results can be reproduced.

DIA proteomic analysis is a relatively new powerful method for investigating protein profiles in ocular disease. This data set pointed to protein categories such as change of cell migration, synapse organization, and cell adhesion in response to intra-ocular SiO. Cadherin 2, transferrin, and lysosome molecules involved in the regulation of cell adhesion, lysosome function, cell death, and iron hemostasis after SO were discovered. These molecules were suggested as indicators of outcome after SiO tamponade in RD. This study gives a deeper understanding of RD and SiO tamponade pathogenesis, which may help to improve the visual outcome after surgery in the future. As an important node, transferrin may provide a research direction to explore the potential mechanism of retinal toxicity of SiO and new treatments to improve the visual function of patients in future studies.

## Data Availability

The datasets presented in this study can be found in online repositories. The names of the repository/repositories and accession number(s) can be found below: http://proteomecentral.proteomexchange.org/cgi/GetDataset PXD026816

## Abbreviations

CAMs, cell adhesion molecules; DIA, data-independent acquisition; DDA, data-dependent acquisition; ELISA, enzyme-linked immunosorbent assay; ERM, epiretinal membrane; ERM group, PPV for epiretinal membrane; FA group, cataract surgery after successful retinal reattachment with sterilized air tamponade; GO, Gene Ontology; iTRAQ, isobaric tags for relative and absolute quantitation; IGF, insulin-like growth factor; KEGG, Kyoto Encyclopedia of Genes and Genomes; LC–MS/MS, liquid chromatography–tandem mass spectrometry; MCL, Markov Cluster Algorithm; PVR, proliferative retinopathy; RRD, rhegmatogenous retinal detachment; RD group, pars plana vitrectomy for rhegmatogenous retinal detachment; SiO, silicone oil; SORVL, silicone oil-related visual loss; SO group, SiO removal after successful retinal reattachment; STRING: Search Tool for the Retrieval of Interacting Genes/Proteins.
