# Supplementary figures and images for: DIA Comparative Proteomic Analysis of Retro-oil Fluid and Vitreous Fluid From Retinal Detachment Patients
Source: Front Mol Biosci. 2021 Dec 3;8:763002. doi: 10.3389/fmolb.2021.763002 (PMC8678117; doi:10.3389/fmolb.2021.763002)

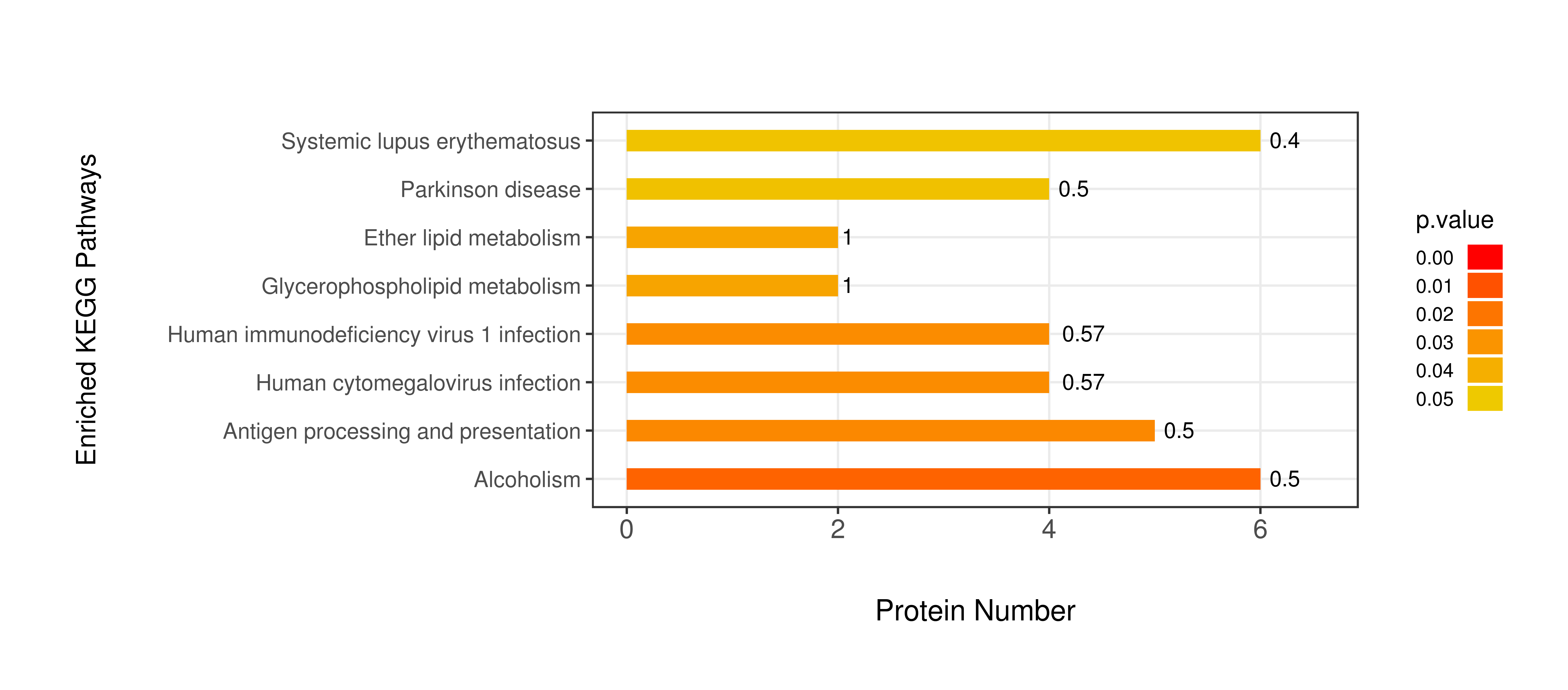

Supplement: Supplementary file 1 [file Image2.PNG]

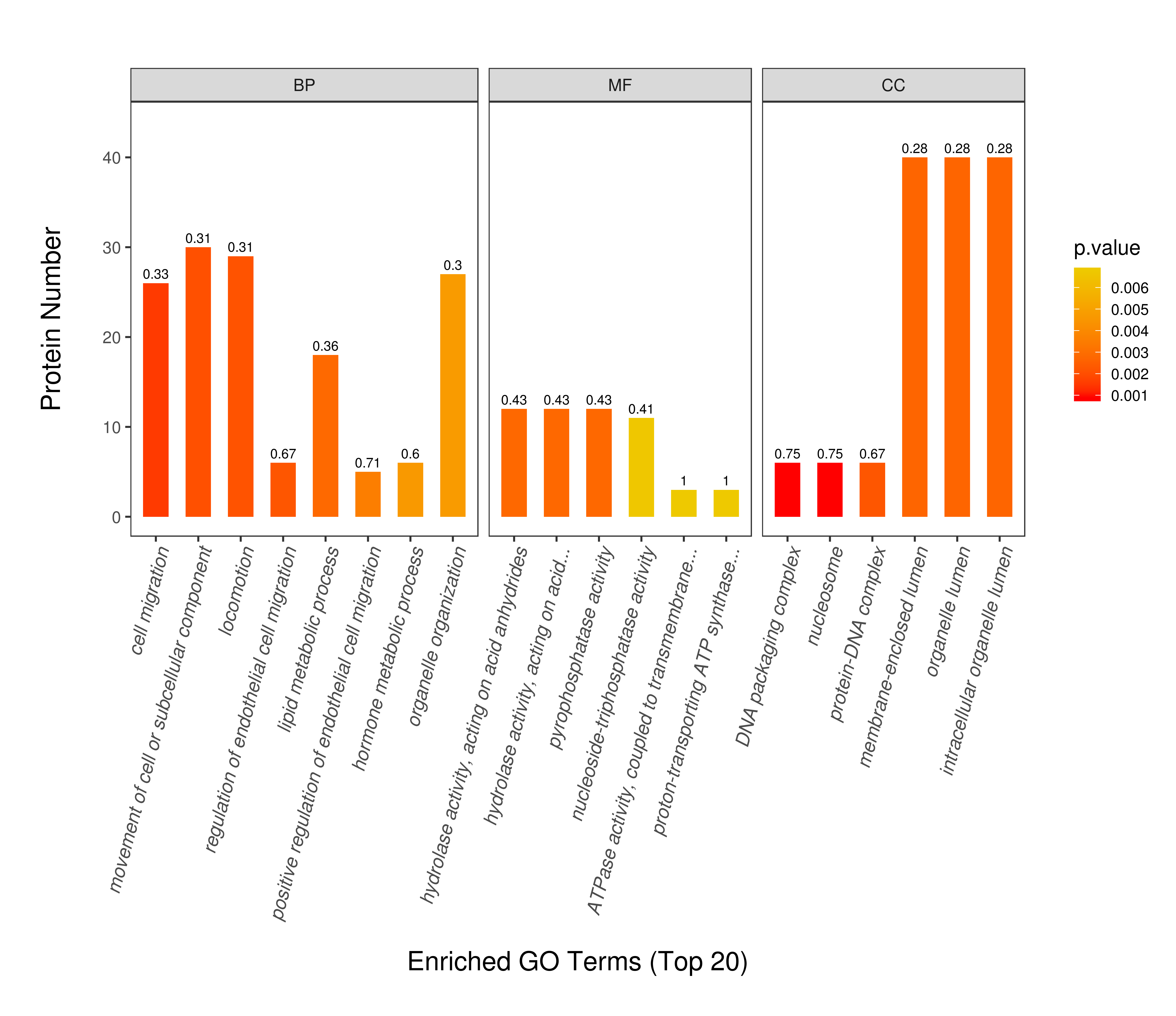

Supplement: Supplementary file 2 [file Image1.PNG]
